# Initial experience with the Codman Certas adjustable valve in the management of patients with hydrocephalus

**DOI:** 10.1186/2045-8118-9-21

**Published:** 2012-09-20

**Authors:** Sara Watt, Niels Agerlin, Bertil Romner

**Affiliations:** 1 The Neuroscience Centre, Department of Neurosurgery, Section NK 2092, Blegdamsvej 9, Copenhagen, DK-2100, Denmark

**Keywords:** Hydrocephalus, Shunt, Certas valve, Adjustable valve, Programmable valve

## Abstract

**Background:**

A new adjustable valve, the Codman Certas^TM^ valve for treatment of hydrocephalus was introduced into clinical practice in January 2011. It has 8 different settings with an opening pressure varying from 36 to over 400 mm H_2_O at a flow rate of 20 mL/h. The 8th setting is designed to provide a "virtual off" function. The objective of this report is to describe the initial clinical experience with the Certas^TM^ valve and evaluate clinical usage with the main focus on the portable adjustment device - Therapeutic Management System (TMS), the “virtual off” setting and compatibility with magnetic resonance imaging (MRI).

**Findings:**

Forty-two patients with hydrocephalus from different etiologies were treated with the Certas^TM^ adjustable shunt system. Data regarding implantation procedures, the use of the TMS system, x-ray imaging, and MRI procedures were recorded prospectively. All patients had clinical follow-up at four weeks after implantation and every three months until a stable clinical condition was obtained.

The mean time for follow-up was 8.6 months (1–16.6). Seventy-one adjustments were performed with the TMS, 12 were problematic. Twenty-nine MRI procedures were performed and did not cause accidental resetting. Five patients were treated with the "virtual off" function for a period.

**Conclusions:**

We found the Certas^TM^ valve valuable in the treatment of hydrocephalus, usability of the TMS was high because it is small and portable, but in some cases we experienced adjustment problems with the first procedures performed by a surgeon, indicating that there is a learning curve. The "virtual off" function provided a possibility of treating over-drainage without the need for shunt ligation or other invasive procedures.

## Introduction

Treating patients with hydrocephalus with an implanted shunt is not always just straightforward and simple: there are occurrences of under-drainage of cerebral spinal fluid (CSF) where a large ventricle and clinical symptoms of hydrocephalus continue or the shunt implantation may cause over-drainage with slit ventricles and development of subdural hygromas [1,2]. It has been shown that adjustable valves can be useful in handling these problems [3-6]. For nearly 25 years, different types of adjustable valves have been on the market with a number of settings from 3 to 20; these valves allow the neurosurgeon to adjust the opening pressure non-invasively and thus correct the volume of CSF shunted. A new adjustable valve, The Codman Certas™ programmable valve, was approved for clinical use in Europe and the USA in 2011. It can be set to 7 different settings for drainage of CSF where the opening pressure for each valve setting varies from 26 mmH_2_O (setting 1) to 247 mmH_2_O (setting 7) at a flow rate of 20 mL/h. An 8th setting is designed to provide a “virtual off” function of the valve, with a mean opening pressure of 494 ± 34 mmH_2_O when flow through the valve should be at a minimum [7].

**Figure 1:**
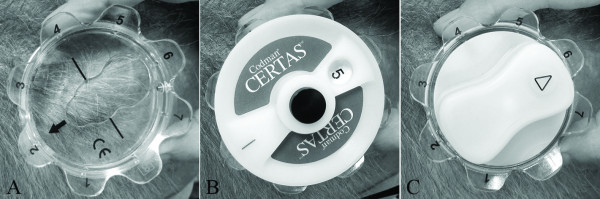
**Therapeutic management system for the Certas**^**TM**^**valve.** Locater tool (**A**), indicator tool (**B**), and adjustment tool (**C**)

The valve system includes a portable adjustment device, the “Therapeutic Management System” (TMS) consisting of a locator, indicator and adjustment tool (Figure 1). Non clinical testing has demonstrated that the Certas^TM^ valve can be considered MRI compatible and can be scanned safely under specific conditions, provided that the static magnetic field is not larger than 3 T [8].

In this report we describe the initial clinical experience with the Certas^TM^ valve and evaluate its clinical value with the main focus on the TMS, “virtual off” setting, and MRI conditionality in the treatment of primary and secondary hydrocephalus in a consecutive series of 42 patients.

## Methods and results

Forty-two patients with hydrocephalus from different etiologies were treated with the Certas^TM^ adjustable shunting system at the Rigshospitalet, Copenhagen (Table 1). Patients were consecutively selected based on etiology of hydrocephalus, in order to include patients with communicating and non communicating hydrocephalus from different etiologies. The patient population consisted of 38% of all shunt implantations performed in the period and mean age was 56 years (5–84), 4 children and 38 adults.

**Table 1:** Etiology of hydrocephalus and the number of patients who had adjustments of the valve setting

**Etiology of hydrocephalus**	**Number of patients**	**Adjusted**
Normal pressure hydrocephalus	14	7
Secondary to subarachnoid hemorrhage	12	7
Congenital	4	3
Secondary to traumatic brain injury	3	3
Secondary to intraventricular hemorrhage	2	1
Secondary to meningitis	2	1
Giant aneurism obstruction	2	0
Tumor obstruction	1	2
Syrinx obstruction	1	1
Aqueductal stenosis	1	0
Total	42	25

There were 22 first shunt implantations and 20 shunt revisions with valve exchange, 26 of the implanted systems included an anti-siphoning device and all implantation procedures were performed by one of 12 specialists in neurosurgery and standardized according to implantation of any ventriculoperitoneal shunt (VP-shunt). All patients had a clinical follow-up 4 weeks after implantation and hereafter every 3 months until the patient was in a stable condition. Data regarding implantation procedures, the use of the TMS system, x-ray imaging, and MRI procedures were recorded prospectively at the time of surgery, during clinical follow-up and at any admissions during the study period.

The mean time of follow-up was 8.6 months (1–16.6); for patients with adjustments of valve settings it was 10.3 (2.6-16.6) while patients with non-adjusted valves had a shorter follow-up of 5.9 (1–16.2) months. The implantation of Codman Certas^TM^ shunt system is a standard procedure similar to implantation of other shunt systems, no surgical revisions related to malfunction of the valve were performed and no patients had verified shunt infections or symptoms relating to this complication.

A total of 71 adjustments were performed with the TMS in 24 patients (Table 2), 12 adjustments were problematic due to difficulties placing indicator or locator tool correctly, resulting in a time consuming adjustment process. The 18 patients who did not have adjustments, had a sufficiently adequate clinical outcome after first follow-up.

**Table 2:** Reasons for performing valve adjustments and number of adjustments

**Reason for adjustment**	**Number of adjustments**
Optimising clinical status due to clinical symptoms of:	
Normal pressure hydrocephalus	10
Over drainage	7
Under drainage	10
CT- results showing large ventricles	9
Hygromas	5
Diminished hygromas	5
Non-specific headache	17
Dizziness	8
Total	71

Twenty-nine cerebral MRI´s were performed in 14 patients (6 at 3.0 T and 23 at 1.5 T), there was no accidental readjustment of the valve setting as verified with TMS and x-ray. The “virtual off” function, setting 8 was used on five occasions due to over-drainage. Three patients developed hygromas prior to implantation of a Certas^TM^ valve after hydrocephalus treatment with fixed shunt types when the previous valve was exchanged for a Certas^TM^ valve with initial setting 8. Two additional patients developed bilateral hygromas after implantation of the Certas^TM^ valve. In all 5 patients setting 8 was maintained for a period until the hygromas had diminished on CT examination. Subsequently the valve setting was gradually decreased. No symptoms of hydrocephalus were detected in any of the patients during treatment with valve setting 8. X-ray verification of the valve setting was performed after MRI and after problematic adjustment procedures. The x-ray procedure for the Certas^TM^ valve was different from other similar valve systems: the cassette is placed on the opposite side of the valve while the camera must be placed on the valve side perpendicular to the valve. The picture quality was not always optimal.

## Discussion

We have presented our first experiences with the new Certas^TM^ valve including clinical advantages or disadvantages with main focus on the TMS, MRI conditionality and the “virtual off” setting.

The follow-up time for patients with adjustments of the valve setting was longer (10.3 months) than follow-up time for non-adjusted patients (5.9 months) because all newly-adjusted patients had a clinical follow-up in the out-patient clinic until the patient was in a stable condition.

We experienced problems performing adjustments with the TMS, mainly resulting in a time-consuming adjustment process, but in three known cases the valve setting was incorrect and led to over drainage and development of hygromas in two patients and severe symptoms in one patient. The problems primarily occurred with the first adjustments procedures performed by an individual surgeon, indicating that there was a learning curve. To avoid adjustment problems in the immediate post-operative period due to swelling, the valve must be tunneled under the intact skin and rest on bone and not in soft neck tissue. To avoid problems due to incorrect placement of locator and indicator tool, it is important to note that the locator tool must follow the valve for the entire length of the valve, as an incorrect position of just millimeters can lead to an incorrect valve setting reading. Furthermore, new users should be introduced to the TMS by an experienced user and trained in the adjustment procedure.

Setting 8, the “virtual off” function is a key function in the Certas^TM^ valve, and in all of the patients where we used setting 8, they avoided one or more invasive procedures that would otherwise have been necessary. We recommend that a specific procedure and instruction for x-ray of Certas^TM^ valve is written and followed. Improvements could also be made in the design of the valve in placement of radiopaque markers to make it easier to verify valve settings on x-ray images. We experienced no accidental change in the valve setting after MRI procedures. MRI compatibility would be of great value and this issue must be investigated further. The valve produced MRI artifacts, but they can be reduced by appropriate selection of pulse sequence parameters [9] and only constitute a problem when the area of interest is very close to the valve. This can be allowed for by siting the valve appropriately at surgery.

At present there are five different adjustable valves on the market (Table 3). The Codman Hakim programmable valve (CHPV) has been described in many studies [3,5,6]. The adjustment system is not portable and accidental readjustments have occurred with exposure to MRI procedures and with exposure to magnetic toys [10,11]. The Strata valve is very similar to the Certas^TM^ valve in design and includes a portable adjustment system quite similar to the TMS. It has also been reported to accidently change settings with exposure to both MRI and magnetic toys [11]. The Sophysa Polaris valve includes a portable adjustment system with a compass tool to indicate correct positioning of indicator tool over the valve [12]. The MIETHKE proGAV® valve includes a gravitational device [13,14]. The Sophysa Polaris and the MIETHKE proGAV valve are both MRI compatible up to 3.0 T but they have no “virtual off” function.

**Table 3:** Relevant individual features of adjustable valves

**Valve**	**CHPV**	**Strata Medtronic**	**Sophysa Polaris**	**Miethke Pro-GAV**	**Certas**
**MRI-safe**	Yes	Yes	Yes	Yes	Yes
**Unintentional adjustments during MRI**	Yes	Yes	No	No	No
**Pressure range (mm H2O)**	30-200	15-135	30-200	0-200	36-400
**Number of settings**	18	5	5*	20	8
**”Virtual off” function**	No	No	No	No	Yes

*Available in 4 different setting intervals with 5 possible settings.

## Conclusions

We found the Certas^TM^ valve valuable in the treatment of hydrocephalus, usability of the TMS was high because it is small and portable, but in some cases we experienced adjustment problems when surgeons were inexperienced. New users should be introduced to the TMS by an experienced user and trained in the adjustment procedure. The “virtual off” function provided the possibility of treating over-drainage without the need for shunt ligation or other invasive procedures. Twenty-nine MRI procedures caused no accidental resetting of the valve, but MRI compatibility needs further investigation.

## Competing interests

There are no competing interests or conflicts of interest for any of the authors, the Codman Company has not had any influence in our work and we have not received any payment of any kind. Bertil Romner is a consultant for the company, but this has not influenced our present report, neither have we received any funding or financial support and the work has been interpreted solely by us and has not been published elsewhere in any form.

## Authors’ contributions

SW contributed to design and planning of the study, acquisition of data, analyzed, interpreted data and drafted the manuscript. NA contributed substantially in the acquisition of data, and was involved in drafting the manuscript. BR contributed to the conception and design of the study, and has critically revised the draft for content and form. All authors have read and approved the final version of the manuscript.
